# Depression and associated factors in medical students in Acapulco during the COVID-19 pandemic: A cross-sectional study

**DOI:** 10.1371/journal.pone.0285903

**Published:** 2023-05-25

**Authors:** Leticia Juanico-Morales, Elizabeth Nava-Aguilera, Arcadio Morales-Pérez, Liliana Morales-Nava, María Atocha Valdez-Bencomo, Abel Emigdio-Vargas, Felipe René Serrano-de los Santos, Neil Andersson

**Affiliations:** 1 Centro de Investigación de Enfermedades Tropicales (CIET), Universidad Autónoma de Guerrero, Acapulco, Guerrero, México; 2 Facultad de Medicina, Universidad Autónoma de Guerrero, Acapulco, Guerrero, México; 3 Jurisdicción Sanitaria 07, Secretaría de Salud, Acapulco, Guerrero, México; 4 Department of Family Medicine, McGill University, Montreal, Quebec, Canada; Goulburn Valley Health, AUSTRALIA

## Abstract

**Background:**

Depression is common in medical students and the Mexican state of Guerrero has the highest rates of depression in the country. Acapulco, the seat of the state medical school, is a tourist destination that experienced early high rates of COVID-19. The COVID-19 pandemic closed all schools in Mexico, obliging a shift from face-to-face to virtual education. In this new context, medical students faced challenges of online teaching including inadequate connectivity and access technologies. Prolonged isolation during the pandemic may have had additional mental health implications.

**Aim:**

Assess depression prevalence and its associated factors affecting medical students in Acapulco, Mexico during the COVID-19 pandemic.

**Methods:**

A cross-sectional survey of students of the Faculty of Medicine of the Universidad Autónoma de Guerrero, in November 2020. After informed consent, students completed a self-administered questionnaire collating socio-demographic, academic and clinical variables, major life events and changes in mood. The Beck inventory provided an assessment of depression. Bivariate and multivariate analyses relied on the Mantel-Haenszel procedure to identify factors associated with depression. We estimated the odds ratio (OR) and 95% confidence intervals.

**Results:**

33.8% (435/1288) of student questionnaires showed evidence of depression in the two weeks prior to the study, with 39.9% (326/817) of young women affected. Factors associated with depression included female sex (OR 1.95; 95%CI 1.48–2.60), age 18–20 years (OR 1.36; 95%CI 1.05–1.77), perceived academic performance (OR 2.97; 95%CI 2.16–4.08), perceived economic hardship (OR 2.18; 95%CI 1.57–3.02), and a family history of depression (OR 1.85; 95%CI 10.35–2.54). Covid-19 specific factors included a life event during the pandemic (OR 1.99; 95%CI 1.54–2.59), connectivity problems during virtual classes and difficulties accessing teaching materials (OR 1.75; 95%CI 1.33–2.30).

**Conclusions:**

The high risk of depression in medical students during the COVID-19 pandemic was associated with perceived academic performance and technical barriers to distance learning, in addition to known individual and family factors. This evidence may be useful for the improvement of programs on prevention and control of depression in university students.

## Introduction

Depression is a common mental disorder that can affect personal activities and relationships with work, school and family [1]. It is a leading cause of disability worldwide, affecting an estimated 280 million people—3.8% of the population [2]. In Mexico, 9.2% of the population has suffered an episode of depression at one or more points in their lives. Guerrero was worse affected among the Mexican states, with 21% of those over 18 years of age reporting symptoms of depression [3].

First year university students are especially vulnerable to depression, separated as they are from their families and familiar environments to take on new lifestyles [4]. Medical students report higher rates of depression than do students from other disciplines [5], possibly because of high pressure learning and exposure to human suffering [6]. A 2016 systematic review placed Mexico at the lower end of depression rates among medical students in 43 countries [7]. In Brazil, 41% of medical students reported depression [8], 30.6% in Cameroon [9], 25% in Chile [10], and 15.2% in Vietnam [11]. Established risk factors for depression in medical students include gender, women are more at risk than men [10, 12–14], age [15], clinical training, concomitant chronic diseases [10], major life events [10, 16], familiar history of depression [15, 16], and perceived academic stress [16].

In March 2020, infection by the coronavirus SARS-CoV-2 (COVID-19), was declared a pandemic [17] causing an historic worldwide interruption of education [18]. Many countries including Mexico adopted school closure as a foundational prevention strategy [19], complementing isolation, social distancing and masking [20, 21]. The prevention combination had consequences beyond the control of infection, not least an increased burden of depression; Mexico registered one of the highest rates of increased depression, the most affected group being young adults aged 20–24 years [22].

Internationally, medical students showed increased rates of depression during the pandemic [23, 24]. The Guerrero state medical school is in Acapulco, a city famously dependent on tourism and that experienced early high rates of COVID-19. This raises a question of what happened to depression risks for medical students in an already high-risk state during the pandemic. With a view to informing support strategies, our aim was to assess depression prevalence and its associated factors affecting medical students in Acapulco during the COVID-19 pandemic.

## Material and methods

### Setting

**Fig 1:**
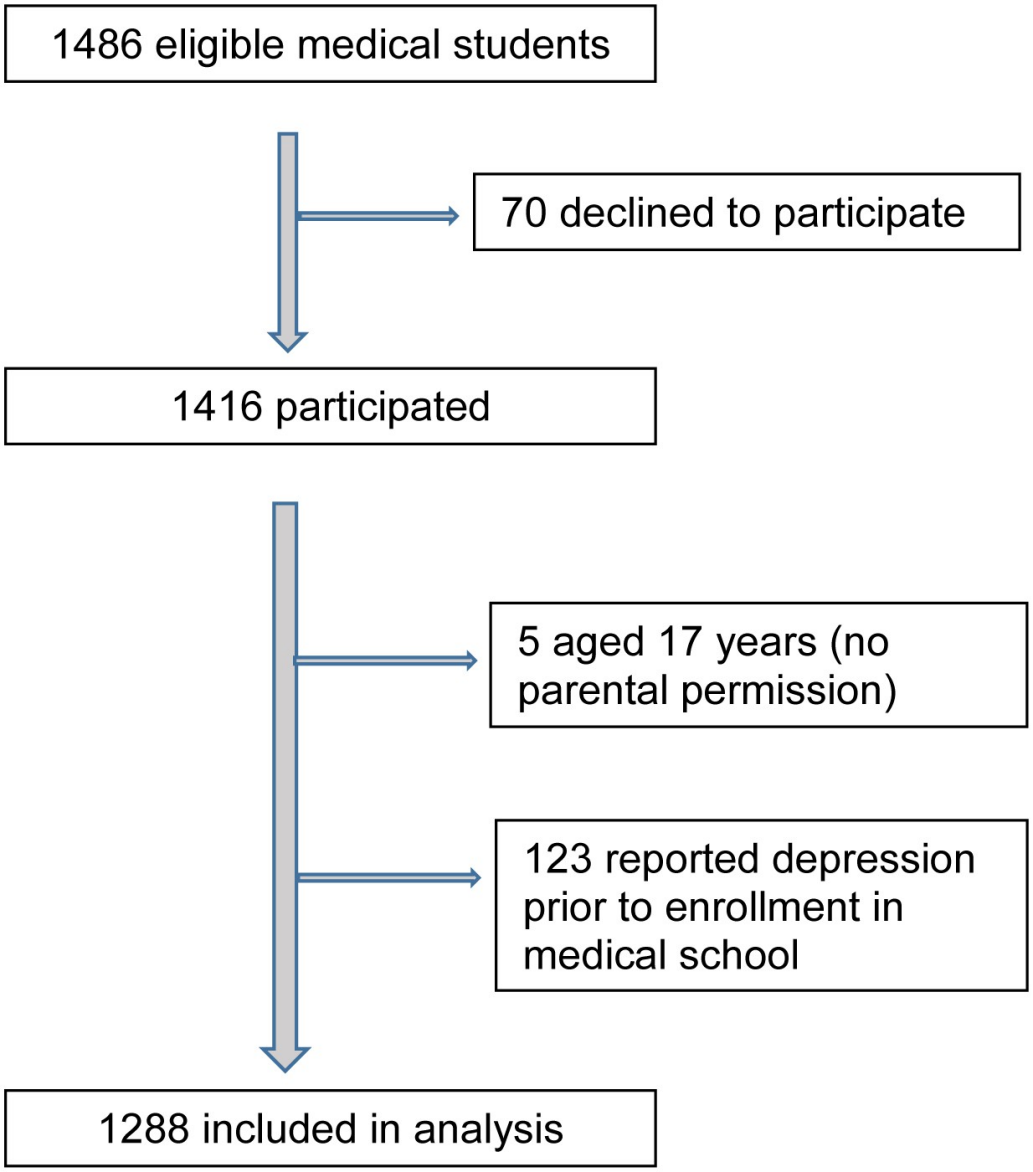
Participant flow diagram.

This cross-sectional study in November 2020 involved students of the Faculty of Medicine of the Universidad Autónoma de Guerrero (UAGro), a large public university with around 90,000 students in one hundred undergraduate and graduate programs. The southern state of Guerrero (population 3.5m) is one of the poorest in Mexico and has a large Indigenous population. The state medical school is in Acapulco, the largest conurbation in the state and a well-known tourist center. The study included all enrolled medical students from first through fourth year who attended classes at the time of the survey. We excluded from the analysis respondents who reported a diagnosis of depression before starting their studies and those under the age of 18 years (Fig 1).

### Measuring instruments

The self-administered questionnaire relied on Google Forms and included 43 items in seven sections: 1) informed consent, 2) sociodemographic data such as school grade, age, sex, marital status, language spoken at home, with whom they lived and perception of their economic situation, 3) academic data on performance, connectivity problems during virtual classes and difficulty in accessing information shared by teachers 4) clinical aspects such as presence of chronic diseases, family history of depression and previous diagnosis of depression, 5) major life events on breakup of love relationship or friendship, traffic accident, hospitalization for serious illness, citizen insecurity, sexual abuse and death of a close relative, 6) changes in mood, and 7) Beck Inventory of depression. The questionnaire used standard questions to cover consent, sociodemographic variables and the Beck Inventory. The research team adapted items on academic, health events, and respondent perceptions from the CIET inventory of household questionnaires [25].

We evaluated depression with the Beck Depression Inventory-II (BDI-II), a self-assessment scale that detects depressive symptoms and quantifies their severity [26, 27]. The 21 items each offer four alternative answers, ordered from least to most severe, for a total score of 0 to 63 points. We asked students to answer each question about how they had felt in the last two weeks, including the day of the survey.

### Operational definition of the main variables

Depression: We considered a student to report depression when the sum of the BDI-II items came to 20 or more. Age and sex referred to years of life completed and self-identified gender.

We derived perception of the current economic situation from the question: How do you perceive your current economic situation? We allowed four answers: excellent, good, average and poor. Perception of current academic performance derived from the question: How do you perceive your current academic performance? We allowed the same four answers: excellent, good, average and poor.

We documented connectivity problems during virtual classes and difficulty accessing documents, presentations or videos shared by teachers by responses to the questions: Have you had connectivity problems during your virtual classes? Have you had difficulties accessing documents, presentations or videos shared by professors? We allowed five answers: never, almost never, occasionally, almost always and always.

We documented experience of a major life event during the COVID-19 pandemic with the question: During the covid-19 pandemic, have you had any major life event or occurrence that has altered your daily activities? We allowed one or more of six answers: breakup of a friendship or love relationship, traffic accident, hospitalization for serious illness, citizen insecurity, sexual abuse or death of a close family member.

We assessed family history of depression with the question: Do you have a family history of depression? We assessed personal history of depression with the question: Before attending medical school, did you have any diagnosis of depression?

### Pilot test

We piloted the self-administered questionnaire in a student body like that in the Faculty of Medicine, after requesting permission from the relevant institutional authorities. Group leaders sent participants the link to the questionnaire in Google Forms via WhatsApp. The pilot assessed clarity and relevance of the questions and estimated the response times for purposes of fieldwork logistics.

### Training for group leaders

We trained the elected student group leaders (one per group of 30–40 students) in a Zoom meeting. Training covered objectives of the study, informed consent, questionnaire content, how to send the web link to their group and the importance of avoiding missing data.

### Data gathering

Before the survey, we requested permission from the medical school authorities. Student group leaders shared the link to the Google Forms questionnaire via WhatsApp. Opening the self-administered questionnaire relied on participants agreeing to provide the information after receiving information about the objectives, confidentiality and anonymity, and their right to decline to answer questions without any academic consequence. They entered responses on-line, with the data stored in the Google cloud.

### Data analysis

An Excel spreadsheet collated responses from the Google platform. We cleaned the data and changed each variable to numerical codes. Analysis relied on the CIETmap statistical software, a Windows-like interface for the popular R code [28]. The unit of analysis was the student. We used the Mantel-Haenszel procedure for bivariate and multivariate analysis [29], expressing the magnitude of effect as an odds ratio (OR) and adjusted odds ratio (aOR). To take account of multiple testing in the bivariate analysis, we computed 99% confidence intervals using the Cornfield method [30]. Following the convention of stepdown procedures in variable selection [31], multivariate analysis began with the saturated model including factors that were statistically significant in the bivariate analysis, stepping down with sequential exclusion of the least significant association, until the final model included only factors significant at the 5% level. We evaluated effect modification with Woolf’s X^2^ squared heterogeneity test [32].

### Ethics statement

The Ethics Committee of the Centro de Investigación de Enfermedades Tropicales at the Universidad Autónoma de Guerrero (UAGro) reviewed and approved the protocol [Approval R-2020-1110-05]. Before fieldwork, we requested and received authorization for the study from the Faculty of Medicine. We informed potential participants of the objectives and benefits of their participation, clarifying that completing the questionnaire was voluntary, anonymous, and confidential. We explained they had the right to decline participation or not to answer any question for any reason, and that they could stop the survey at any time. As we held no record of who had started or finished the questionnaire, participation could not affect their academic positions. We explained that initiating the questionnaire represented informed consent to join the study. The Institutional Review Board waived the need for parental informed consent for those under the age of majority (18 years in Mexico).

## Results

The survey included 95% (1416/1486) of those regularly attending classes. The remaining 70 declined to participate and we have no details on how they differed from the respondents. We excluded 123 respondents who reported a diagnosis of depression before entering university and five potential participants aged under 18 years. The analysis thus included 1288 students (Fig 1) of whom 52.6% (677/1288) in their first four semesters of the eight-semester plan of studies. The mean age was 21 ± 2.3 years; 63.4% (817/1288) of respondents were women. 19.6% (252/1288) reported a family history of depression. Table 1 shows participant characteristics.

**Table 1:** Characteristics of participants.

Variable	Total	1st year Semesters 1 and 2	2nd year Semesters 3 and 4	3rd year Semesters 5 and 6	4th year Semesters 7 and 8
**Age**	n = 1288	n = 375	n = 302	n = 311	n = 300
18	102 (7.9%)	100 (26.7%)	2 (0.7%)	0 (0%)	0 (0%)
19	229 (17.8%)	146 (38.9%)	82 (27.2%)	1 (0.3%)	0 (0%)
20	235 (18.2%)	67 (17.9%)	106 (35.1%)	61 (19.6%)	1 (0.3%)
≥ 21 years	722 (56.1%)	62 (16.5%)	112 (37.1%)	249 (80.1%)	299 (99.7%)
**Sex**					
Female	817 (63.4%)	253 (67.5%)	178 (58.9%)	195 (62.7%)	191 (63.7%)
Male	471 (36.6%)	122 (32.5%)	124 (41.1%)	116 (37.3%)	109 (36.3%)
**Marital status**					
Single	1249 (97%)	367 (97.9%)	294 (97.4%)	305 (98.1%)	283 (94.3%)
Free union, Married, Divorced, Widowed	39 (3%)	8 (2.1%)	8 (2.6%)	6 (1.9)	17 (5.7%)
**Languages spoken at home other than Spanish**					
Indigenous language	103 (8%)	25 (6.7%)	30 (9.9%)	28 (9%)	20 (6.7%)
Spanish	1185 (92%)	350 (93.3)	272 (90.1%)	283 (91%)	280 (93.3%)
**People with whom they live**					
Friends, Partner, Alone, Other	179 (13.9%)	38 (10.1%)	31 (10.3%)	37 (11.9%)	73 (24.3%)
Parents	1109 (86.1%)	337 (89.9%)	271 (89.7%)	274 (88.1%)	227 (75.7%)
**Perception of self-economic situation prior to COVID-19 pandemic**					
Excellent	89 (6.9%)	20 (5.3%)	26 (8.6%)	23 (7.4%)	20 (6.7%)
Good	623 (48.4%)	197 (52.5%)	133 (44%)	149 (47.9%)	144 (48%)
Average	544 (42.2%)	153 (40.8%)	134 (44.4%)	132 (42.4%)	125 (41.7%)
Poor	32 (2.5%)	5 (1.3%)	9 (3.0%)	7 (2.3%)	11 (3.7%)
**Perception of current self-economic situation**					
Excellent	39 (3.0%)	11 (2.9%)	12 (4.0%)	10 (3.2%)	6 (2.0%)
Good	331 (25.7%)	97 (25.9%)	66 (21.9%)	85 (27.3%)	83 (27.7%)
Average	681 (52.9%)	209 (55.7%)	153 (50.7%)	170 (54.7%)	149 (49.7%)
Poor	237 (18.4%)	58 (15.5%)	71 (23.5%)	46 (14.8%)	62 (20.7%)
**Perception of academic performance prior to COVID-19 pandemic**					
Excellent	225 (17.5%)	112 (29.9%)	35 (11.6%)	33 (10.6%)	45 (15.0%)
Good	881 (68.4%)	230 (61.3%)	223 (73.8%)	208 (66.9%)	220 (73.3%)
Average	173 (13.4%)	33 (8.8%)	43 (14.2%)	65 (20.9%)	32 (10.7%)
Poor	9 (0.7%)	0 (0.0%)	1 (0.3%)	5 (1.6%)	3 (1%)
**Perception of current academic performance**					
Excellent	29 (2.3%)	7 (1.9%)	6 (2.0%)	7 (2.3%)	9 (3%)
Good	361 (28.0%)	100 (26.7%)	65 (21.5%)	97 (31.2%)	99 (33%)
Average	693 (53.8%)	204 (54.4%)	182 (60.3%)	160 (51.4%)	147 (49%)
Poor	205 (15.9%)	64 (17.1%)	49 (16.2%)	47 (15.1%)	45 (15%)
**Connectivity problems and difficulty accessing documents, presentations or videos shared by teachers**					
Almost always or always	377 (29.3%)	102 (27.2%)	94 (31.1%)	93 (29.9%)	88 (29.3%)
Occasionally, rarely or never	911 (70.7%)	273 (72.8%)	208 (68.9%)	218 (70.1%)	212 (70.7%)
**Suffers from chronic disease**					
Yes	50 (3.9%)	15 (4.0%)	11 (3.6%)	16 (5.1%)	8 (2.7%)
No	1238 (96.1%)	360 (96.0%)	291 (96.4%)	295 (94.9%)	292 (97.3%)
**Experienced a major life event during the COVID-19 pandemic**					
Yes	548 (42.5%)	160 (42.7%)	124 (41.1%)	143 (46%)	121 (40.3%)
No	740 (57.5%)	215 (57.3%)	178(58.9%)	168 (54%)	179 (59.7%)
**Changes in mood during the COVID-19 pandemic**					
Yes	1072 (83.2%)	331 (88.3%)	249 (82.5%)	258 (83%)	234 (78%)
No	216 (16.8%)	44 (11.7%)	53 (17.5%)	53 (17.0%)	66 (22%)
**Family history of depression**					
Yes	252 (19.6%)	65 (17.3%)	57 (18.9%)	72 (23.2%)	58 (19.3%)
No	1036 (80.4%)	310 (82.7%)	245 (81.1%)	239 (76.8%)	242 (80.7%)

Among students who had not received a diagnosis of depression before enrolling in medical school, 33.8% (435/1288) reported depression in the last two weeks, and 83.2% (1072/1288) reported mood changes over this period. Among young women, 39.9% (326/817) reported depression. Traumatic events affected a large proportion of students in the course of the pandemic: 42.5% (548/1288) reported a life event, of whom 47.3% (259/548) reported death of family or friends; 19.5% (107/548) breakup of friendship/love relationship; 10.2% (56/548) hospitalization for serious illness; 7.3% (40/548) citizen insecurity; 5.1% (28/548) personal problems; 4.2% (23/548) had family problems; 3.3% (18/548) became ill; 2.3% (13/548) experienced traffic accidents; 0.4% (2/548) attempted suicide; and 0.4% (2/548) suffered sexual abuse.

Table 2 shows bivariate analysis of factors associated with depression. Medical students were more likely to present depression if they were: aged 18 to 20 years, female sex, in the first four of eight semesters, perceived economic hardship before the pandemic, perceived academic performance before the pandemic, current Grade Point Average (GPA), and family history of depression. Depression was also associated with pandemic-specific factors including perceived current economic hardship, perceived current academic performance, connectivity problems during virtual classes and difficulty accessing documents, presentations, or videos shared by teachers; a major life event during the pandemic, and changes in mood during the COVID-19 pandemic.

**Table 2:** Bivariate analysis of factors associated with depression in Acapulco medical students (number with and without depression, percentage, odds of experiencing depression and 99% confidence interval).

Factors	Depression		%	OR	99%CI
	Yes	No			
**Year of study**					
Semester 1–4 (year 1–2)	253	424	37.4	**1.41**	**1.03–1.93**
Semester 5–8 (year 3–4)	182	429	29.8		
**Age**					
18 to 20 years	213	352	37.6	**1.36**	**1.0–1.86**
≥ 21 years	222	500	30.7		
**Sex**					
Female	326	491	39.9	**2.21**	**1.58–3.17**
Male	109	362	23.1		
**Marital status**					
Single	420	829	33.6	0.81	0.33–2.47
Free union, Married, Divorced, Widowed	15	24	38.5		
**Languages spoken at home other than Spanish**					
Indigenous language	45	58	43.7	1.58	0.88–2.75
Spanish	390	795	32.9		
**People with whom they live**					
Friends, Partner, Alone, Other	63	116	35.2	1.08	0.67–1.66
Parents	372	737	33.5		
**Perception of self-economic situation prior to COVID-19 pandemic**					
Average, Poor	235	341	40.8	**1.76**	**1.29–2.42**
Excellent, Good	200	512	28.1		
**Perception of current self-economic situation**					
Average, Poor	368	550	40.1	**3.03**	**2.08–4.70**
Excellent, Good	67	303	18.1		
**Perception of academic performance prior to COVID-19 pandemic**					
Average, Poor	79	103	43.4	**1.62**	**1.04–2.48**
Excellent, Good	356	750	32.2		
**Perception of current academic performance**					
Average, Poor	378	520	42.1	**4.25**	**2.89–6.82**
Excellent, Good	57	333	14.6		
**Current Grade Point Average (GPA)**					
≤8	157	187	45.6	**2.01**	**1.43–2.84**
>8	278	666	29.4		
**Grade point average prior to the COVID-19 pandemic**					
≤8	97	170	36.3	1.15	0.78–1.67
>8	338	683	33.1		
**Connectivity problems and difficulty accessing documents, presentations, or videos**					
Almost always, Always	182	195	48.3	**2.43**	**1.74–3.40**
Never, Hardly ever, Occasionally	253	658	27.8		
**Suffers from chronic disease**					
Yes	26	24	52	2.20	0.97–5.09
No	409	829	33.1		
**Experienced a major life event during the COVID-19 pandemic**					
Yes	245	303	45	**2.34**	**1.71–3.23**
No	190	550	25.6		
**Changes in mood during the COVID-19 pandemic**					
Yes	423	649	39.5	**11.08**	**5.85–47.70**
No	12	204	5.6		
**Family history of depression**					
Yes	123	129	49.4	**2.21**	**1.51–3.24**
No	312	724	30.1		

In the multivariate analysis (Table 3), seven factors remained significantly associated with depression: perception of academic performance, perception of economic hardship, a major life event during the pandemic, female sex, having a family history of depression, having connectivity problems during virtual classes and difficulty accessing educational materials shared by teachers, and being 18 to 20 years of age. Factors dropped from the saturated model were: Semester, perception of the economic situation prior to COVID-19 pandemic, perception of academic performance prior to the pandemic, and current GPA.

**Table 3:** Multivariate analysis of factors associated with depression in Acapulco medical students (unadjusted odds ratio, adjusted odds ratio and 95% confidence intervals).

Factors	cOR	aOR	95%aCI
Perception of current academic performance (average /poor)	3.87	2.97	2.16–4.08
Perception of current self-economic situation (average/ poor)	2.75	2.18	1.57–3.02
Experienced a major life event during the COVID-19 pandemic	2.51	1.99	1.54–2.57
Sex (female)	2.05	1.95	1.47–2.59
Family history of depression	1.97	1.85	1.35–2.54
Experienced connectivity problems during virtual classes and difficulty accessing documents, presentations, or videos shared by teachers	2.28	1.75	1.33–2.30
Age 18 to 20 years (younger students)	1.30	1.36	1.05–1.77

## Discussion

We found widespread evidence of depression in medical students in Acapulco, a high-COVID-19 risk city in Guerrero, a high depression risk Mexican state, during the pandemic. The self-administered questionnaire identified one in every three medical students (33.8%) with depression during the two weeks before a survey during the pandemic, young women being at higher risk than men. Excluding those who diagnosed with depression before enrollment in medical school, the non-pandemic factors independently associated with depression included younger age, family history of depression. Independent of these factors, pandemic-related associations included a major life event during the pandemic, perception of average/poor current academic performance, perception of average/poor current economic situation, problems with connectivity during virtual classes and access to educational materials.

The two-week period prevalence of depression in medical students in our study was within the range reported in the literature [7, 33], somewhat lower than that reported by Guerrero *et al*. (39.3%) [34], Brenneisen *et al*. (41%) [8], and Al Saadi *et al*. (60.6%) [12], but higher than many other studies [7, 8, 35–37]. The demanding curriculum [38], exam pressure [39], lack of time for leisure activities, and social interaction [40] help to explain depression in medical students. During their clinical years, medical students face added challenges in patient care [41]. Added to the “usual” stress of medical studies, COVID-19 has had negative effects on student life [42]. Our finding of an association between depression and perceived poor academic performance could be part of this dynamic, as could the finding of an association with the students perceived economic situation. Not being able to take part in social activities that involve monetary cost [43] could provoke symptoms of anxiety, sadness and depression [44, 45].

The association we found between depression and perceived academic performance fits with findings of Bostanci *et al*., who reported that students with poor school performance are more likely to have depressive symptoms [46]. Romo *et al*. reported students with high levels of perceived academic stress were more likely to show symptoms of depression [16]. Our finding of an association between depression and perception of economic hardship was also the focus of Al Saadi *et al*. and Bostanci *et al*., who found students who reported their personal income as intermediate or insufficient were more likely to report depression [12, 46].

We found major life events during the COVID-19 pandemic associated with depression. This echoes findings of Ngasa *et al*. [9] and Kebede *et al*. [15]. There is a recognized association between depression and major life events such as relationship breakup, hospitalization for serious illness and death of a family member [47]. The individual response to this type of stressful events is variable but could contribute to depressive reactions [48]. The current health crisis could only have accentuated these problems. Social distancing to reduce spread of SARS-CoV2 could affect relationships with friends and family. Hospitalization or death of loved ones, unaccompanied by people close to them, cause pain, guilt, sadness, and can affect mental health [49].

Our finding that depression was more likely to affect young women is consistent with other reports [9, 13–15]. The association between gender and depression is compatible with young women being more likely to report insomnia, recurrent pain, sadness, nervousness, anxiety, anguish and depression [50]. Women are also more likely to report physical or emotional ailments, a trend that increases with the number of symptoms they experience [51].

Our finding of a family history of depression is consistent with those of Al Maashani *et al*. [14], and could derive from different dynamics [52]. One candidate mechanism is that depression during pregnancy and the transmission of the mother’s cognitive styles might contribute to the development of depression in the offspring [53].

Connectivity problems during virtual classes and difficult access to documents, presentations or videos shared by teachers was more common among students with depression. We found no previous reports of this association although Al Zahrani *et al*.; reported that 41.8% of health sciences students considered virtual learning as a stressful experience [54]. A pandemic-specific problem was the association between depression and connectivity problems during virtual classes. Guerrero State has poorly developed telecommunications infrastructure, precipitating network connection interruptions during classes. This requires repeated reconnection to sessions, loss of continuity and information, and reduced opportunity to take exams in a timely manner. These technological barriers produce stress, anxiety and depressive symptoms in the student [55].

We found younger medical students (18 to 20 years of age) more likely to report depression. This supports the finding of Kebede *et al*. [15] and goes against the results of Wafaa *et al*. who found age over 20 years was a predictor of depression [56]. In our case, younger students are in their first years of college and are going through a period of adaptation to university life [57]. This leads to changes in lifestyle and time management [58]. As a result, they might feel unable to cope with the extensive curriculum [59]. Pressure to learn new concepts might lead to anxiety, stress and depression [16].

Our results confirm that depression is a genuine problem in medical students and echoes earlier calls for prevention and control strategies, including psychological care programs offered by universities [60, 61] and healthcare institutions [62]. Ideally these programs should engage students, professors, tutors, school management and the health institutions with responsibility to promote the mental health [63]. Potential actions include peer support networks that allow the establishment of bonds of trust, and workshops for stress management and emotional control [64]. A student financial support program would help those students whose head of the family suffered unemployment, lengthy illness, or death because of the COVID-19 pandemic [65].

### Limitations and strengths

Without comparable measurement in this population before the pandemic, we cannot say how much of the depression is due to the pandemic or even if there was an increase during the pandemic. Another limitation, common to most cross-sectional studies, is temporality of the associated factors in relation to depression: we are unable to say, for example, whether the perceived academic performance and economic situation preceded the depression or followed it.

Self-reporting of the Beck Depression Inventory-II items is a limitation shared with other questionnaire-based studies of depression. The many items in the Inventory should reduce the effects of random answers but there was no independent clinical verification by a qualified psychologist or psychiatrist. We excluded 123 students who reported a diagnosis of depression before entering medical school and we asked perception about academic performance before the pandemic and at the time of the survey, obtaining information eight months after the onset of the COVID-19 pandemic.

We are aware of the limits in generalizability of our findings. The UAGro Faculty of Medicine is part of a large public and autonomous institution of higher education in the state of Mexico’s Guerrero State. Acapulco is one of the country’s main tourist destinations and economic centers, exposing 23% of the state’s population [66] to any increased risk of infection accompanying the pandemic.

A strength of our study includes the high response rate of 95% (1416/1494). This may be due to our meeting with group leaders before the survey and their training on how to distribute the survey web link to their classmates. Previous studies on depression in students report response rates of 75% to 90% [9, 34, 57].

## Conclusion

The study confirms a high risk of depression in medical students in a high COVID risk city in a high depression risk state, based on a high response rate (95%). It confirms already recognized risk factors (sex, age, perceived academic performance and economic difficulties) and implicates COVID-specific factors related to distance learning and connectivity. Complementing studies in other settings that draw attention to depression in medical students, educational authorities and teachers might find this information useful in prevention, diagnosis, and treatment strategies.

## Supporting information

S1 Checklist STROBE statement—Checklist of items that should be included in reports of *cross-sectional studies*. (DOC)

S1 Data Dataset depression. (CSV)

S2 Data Database coding sheet of “Depression and associated factors in medical students in Acapulco during the COVID-19 pandemic: A cross-sectional study”. (DOCX)

S1 Table Tables depression v10academi v8escurren v15event v3sex v17fhd v13connect v2age. (DOCX)
