# Supplementary material for: Depression and associated factors in medical students in Acapulco during the COVID-19 pandemic: A cross-sectional study
Source: PLoS One. 2023 May 25;18(5):e0285903. doi: 10.1371/journal.pone.0285903 (PMC10212175; doi:10.1371/journal.pone.0285903)
Supplement: S2 Data — (DOCX) [file pone.0285903.s003.docx]

**Database coding sheet of “Depression and associated factors in medical students in Acapulco during the COVID-19 pandemic: a cross-sectional study”**

**Dataset Depression_MS.csv**

| **Variables** | **Variables name** | **Code** |
| --- | --- | --- |
| Depression | depression | 1= Yes  2= No |
| Semester attender | v1Semester | 1= First through fourth semester  2= Fifth through eighth semester |
| Age | v2Age | 1= 18-20 años  2= 21-45 |
| Sex | v3Sex | 1 = Female  2 = Male |
| Marital status | v4Mstatus | 1= Single  2= Married, Free union, Divorced, Widowed |
| Languages spoken at home other than Spanish | v5Lspoken | 1=Ñomnda, Na zavii, Náhuatl, Mephaa  2= Spanish exclusively |
| People with whom they live | v6People | 1= Friends, Partner, Alone, Other  2= Parents |
| Perception of self-economic situation prior to COVID-19 pandemic | v7ESprior | 1= Average, Poor  2= Excellent, Good |
| Perception of current self-economic situation | v8EScurrent | 1= Average, Poor  2= Excellent, Good |
| Perception of academic performance prior to COVID-19 pandemic | v9Academic | 1= Average, Poor  2= Excellent, Good |
| Perception of current academic performance | v10Academic | 1= Average, Poor  2= Excellent, Good |
| Current Grade Point Average (GPA) | v11GPA | 1 ≤8  2 >8 |
| Grade point average prior to COVID-19 pandemic | v12GPA | 1 ≤8  2 >8 |
| Experienced connectivity problems during virtual classes and difficulty accessing documents, presentations  or videos shared by teachers | V13Connect | 1= Almost always, Always  2= Never, Hardly ever, Occasionally |
| Suffers from chronic disease | v14Chronic | 1= Yes  2= No |
| Experienced a major life event  during the COVID-19 pandemic | v15Event | 1= Yes  2= No |
| Changes in mood during the  COVID-19 pandemic | v16Mood | 1= Yes  2= No |
| Family history of depression | v17FHD | 1= Yes  2= No |
