# Supplementary material for: Depression and associated factors in medical students in Acapulco during the COVID-19 pandemic: A cross-sectional study
Source: PLoS One. 2023 May 25;18(5):e0285903. doi: 10.1371/journal.pone.0285903 (PMC10212175; doi:10.1371/journal.pone.0285903)
Supplement: S1 Table — (DOCX) [file pone.0285903.s004.docx]

Tables depression v10academi v8escurren v15event v3sex v17fhd v13connect v2age

selection: all records selected

| V10ACADEMI |

DEPRESSION | 1.0 2.0 | Total

-----------+---------------+-------

1.0 | 378 57 | 435

> 86.9% 13.1% | 33.8%

| 42.1% 14.6% |

2.0 | 520 333 | 853

> 61.0% 39.0% | 66.2%

| 57.9% 85.4% |

-----------+---------------+-------

Total | 898 390 | 1288

| 69.7% 30.3% |

V8ESCURREN = 1.0

V15EVENT = 1.0

V3SEX = 1.0

V17FHD = 1.0

V13CONNECT = 1.0

V2AGE = 1.0

| V10ACADEMI |

DEPRESSION | 1.0 2.0 | Total

-----------+---------------+-------

1.0 | 9 1 | 10

> 90.0% 10.0% | 83.3%

| 81.8% 100.0% |

2.0 | 2 0 | 2

> 100.0% 0.0% | 16.7%

| 18.2% 0.0% |

-----------+---------------+-------

Total | 11 1 | 12

| 91.7% 8.3% |

V8ESCURREN = 1.0

V15EVENT = 1.0

V3SEX = 1.0

V17FHD = 1.0

V13CONNECT = 1.0

V2AGE = 2.0

| V10ACADEMI |

DEPRESSION | 1.0 2.0 | Total

-----------+---------------+-------

1.0 | 14 2 | 16

> 87.5% 12.5% | 69.6%

| 77.8% 40.0% |

2.0 | 4 3 | 7

> 57.1% 42.9% | 30.4%

| 22.2% 60.0% |

-----------+---------------+-------

Total | 18 5 | 23

| 78.3% 21.7% |

V8ESCURREN = 1.0

V15EVENT = 1.0

V3SEX = 1.0

V17FHD = 1.0

V13CONNECT = 2.0

V2AGE = 1.0

| V10ACADEMI |

DEPRESSION | 1.0 2.0 | Total

-----------+---------------+-------

1.0 | 11 0 | 11

> 100.0% 0.0% | 68.8%

| 91.7% 0.0% |

2.0 | 1 4 | 5

> 20.0% 80.0% | 31.3%

| 8.3% 100.0% |

-----------+---------------+-------

Total | 12 4 | 16

| 75.0% 25.0% |

V8ESCURREN = 1.0

V15EVENT = 1.0

V3SEX = 1.0

V17FHD = 1.0

V13CONNECT = 2.0

V2AGE = 2.0

| V10ACADEMI |

DEPRESSION | 1.0 2.0 | Total

-----------+---------------+-------

1.0 | 12 0 | 12

> 100.0% 0.0% | 42.9%

| 54.5% 0.0% |

2.0 | 10 6 | 16

> 62.5% 37.5% | 57.1%

| 45.5% 100.0% |

-----------+---------------+-------

Total | 22 6 | 28

| 78.6% 21.4% |

V8ESCURREN = 1.0

V15EVENT = 1.0

V3SEX = 1.0

V17FHD = 2.0

V13CONNECT = 1.0

V2AGE = 1.0

| V10ACADEMI |

DEPRESSION | 1.0 2.0 | Total

-----------+---------------+-------

1.0 | 21 2 | 23

> 91.3% 8.7% | 69.7%

| 72.4% 50.0% |

2.0 | 8 2 | 10

> 80.0% 20.0% | 30.3%

| 27.6% 50.0% |

-----------+---------------+-------

Total | 29 4 | 33

| 87.9% 12.1% |

V8ESCURREN = 1.0

V15EVENT = 1.0

V3SEX = 1.0

V17FHD = 2.0

V13CONNECT = 1.0

V2AGE = 2.0

| V10ACADEMI |

DEPRESSION | 1.0 2.0 | Total

-----------+---------------+-------

1.0 | 26 4 | 30

> 86.7% 13.3% | 53.6%

| 53.1% 57.1% |

2.0 | 23 3 | 26

> 88.5% 11.5% | 46.4%

| 46.9% 42.9% |

-----------+---------------+-------

Total | 49 7 | 56

| 87.5% 12.5% |

V8ESCURREN = 1.0

V15EVENT = 1.0

V3SEX = 1.0

V17FHD = 2.0

V13CONNECT = 2.0

V2AGE = 1.0

| V10ACADEMI |

DEPRESSION | 1.0 2.0 | Total

-----------+---------------+-------

1.0 | 21 1 | 22

> 95.5% 4.5% | 40.0%

| 48.8% 8.3% |

2.0 | 22 11 | 33

> 66.7% 33.3% | 60.0%

| 51.2% 91.7% |

-----------+---------------+-------

Total | 43 12 | 55

| 78.2% 21.8% |

V8ESCURREN = 1.0

V15EVENT = 1.0

V3SEX = 1.0

V17FHD = 2.0

V13CONNECT = 2.0

V2AGE = 2.0

| V10ACADEMI |

DEPRESSION | 1.0 2.0 | Total

-----------+---------------+-------

1.0 | 23 9 | 32

> 71.9% 28.1% | 45.1%

| 45.1% 45.0% |

2.0 | 28 11 | 39

> 71.8% 28.2% | 54.9%

| 54.9% 55.0% |

-----------+---------------+-------

Total | 51 20 | 71

| 71.8% 28.2% |

V8ESCURREN = 1.0

V15EVENT = 1.0

V3SEX = 2.0

V17FHD = 1.0

V13CONNECT = 1.0

V2AGE = 1.0

| V10ACADEMI |

DEPRESSION | 1.0 2.0 | Total

-----------+---------------+-------

1.0 | 3 0 | 3

> 100.0% 0.0% | 75.0%

| 75.0% -1.$% |

2.0 | 1 0 | 1

> 100.0% 0.0% | 25.0%

| 25.0% -1.$% |

-----------+---------------+-------

Total | 4 0 | 4

| 100.0% 0.0% |

V8ESCURREN = 1.0

V15EVENT = 1.0

V3SEX = 2.0

V17FHD = 1.0

V13CONNECT = 1.0

V2AGE = 2.0

| V10ACADEMI |

DEPRESSION | 1.0 2.0 | Total

-----------+---------------+-------

1.0 | 2 1 | 3

> 66.7% 33.3% | 60.0%

| 50.0% 100.0% |

2.0 | 2 0 | 2

> 100.0% 0.0% | 40.0%

| 50.0% 0.0% |

-----------+---------------+-------

Total | 4 1 | 5

| 80.0% 20.0% |

V8ESCURREN = 1.0

V15EVENT = 1.0

V3SEX = 2.0

V17FHD = 1.0

V13CONNECT = 2.0

V2AGE = 1.0

| V10ACADEMI |

DEPRESSION | 1.0 2.0 | Total

-----------+---------------+-------

1.0 | 2 2 | 4

> 50.0% 50.0% | 28.6%

| 28.6% 28.6% |

2.0 | 5 5 | 10

> 50.0% 50.0% | 71.4%

| 71.4% 71.4% |

-----------+---------------+-------

Total | 7 7 | 14

| 50.0% 50.0% |

V8ESCURREN = 1.0

V15EVENT = 1.0

V3SEX = 2.0

V17FHD = 1.0

V13CONNECT = 2.0

V2AGE = 2.0

| V10ACADEMI |

DEPRESSION | 1.0 2.0 | Total

-----------+---------------+-------

1.0 | 5 1 | 6

> 83.3% 16.7% | 46.2%

| 45.5% 50.0% |

2.0 | 6 1 | 7

> 85.7% 14.3% | 53.8%

| 54.5% 50.0% |

-----------+---------------+-------

Total | 11 2 | 13

| 84.6% 15.4% |

V8ESCURREN = 1.0

V15EVENT = 1.0

V3SEX = 2.0

V17FHD = 2.0

V13CONNECT = 1.0

V2AGE = 1.0

| V10ACADEMI |

DEPRESSION | 1.0 2.0 | Total

-----------+---------------+-------

1.0 | 16 0 | 16

> 100.0% 0.0% | 76.2%

| 76.2% -1.$% |

2.0 | 5 0 | 5

> 100.0% 0.0% | 23.8%

| 23.8% -1.$% |

-----------+---------------+-------

Total | 21 0 | 21

| 100.0% 0.0% |

V8ESCURREN = 1.0

V15EVENT = 1.0

V3SEX = 2.0

V17FHD = 2.0

V13CONNECT = 1.0

V2AGE = 2.0

| V10ACADEMI |

DEPRESSION | 1.0 2.0 | Total

-----------+---------------+-------

1.0 | 4 3 | 7

> 57.1% 42.9% | 38.9%

| 28.6% 75.0% |

2.0 | 10 1 | 11

> 90.9% 9.1% | 61.1%

| 71.4% 25.0% |

-----------+---------------+-------

Total | 14 4 | 18

| 77.8% 22.2% |

V8ESCURREN = 1.0

V15EVENT = 1.0

V3SEX = 2.0

V17FHD = 2.0

V13CONNECT = 2.0

V2AGE = 1.0

| V10ACADEMI |

DEPRESSION | 1.0 2.0 | Total

-----------+---------------+-------

1.0 | 4 0 | 4

> 100.0% 0.0% | 28.6%

| 30.8% 0.0% |

2.0 | 9 1 | 10

> 90.0% 10.0% | 71.4%

| 69.2% 100.0% |

-----------+---------------+-------

Total | 13 1 | 14

| 92.9% 7.1% |

V8ESCURREN = 1.0

V15EVENT = 1.0

V3SEX = 2.0

V17FHD = 2.0

V13CONNECT = 2.0

V2AGE = 2.0

| V10ACADEMI |

DEPRESSION | 1.0 2.0 | Total

-----------+---------------+-------

1.0 | 5 0 | 5

> 100.0% 0.0% | 20.0%

| 29.4% 0.0% |

2.0 | 12 8 | 20

> 60.0% 40.0% | 80.0%

| 70.6% 100.0% |

-----------+---------------+-------

Total | 17 8 | 25

| 68.0% 32.0% |

V8ESCURREN = 1.0

V15EVENT = 2.0

V3SEX = 1.0

V17FHD = 1.0

V13CONNECT = 1.0

V2AGE = 1.0

| V10ACADEMI |

DEPRESSION | 1.0 2.0 | Total

-----------+---------------+-------

1.0 | 2 1 | 3

> 66.7% 33.3% | 37.5%

| 28.6% 100.0% |

2.0 | 5 0 | 5

> 100.0% 0.0% | 62.5%

| 71.4% 0.0% |

-----------+---------------+-------

Total | 7 1 | 8

| 87.5% 12.5% |

V8ESCURREN = 1.0

V15EVENT = 2.0

V3SEX = 1.0

V17FHD = 1.0

V13CONNECT = 1.0

V2AGE = 2.0

| V10ACADEMI |

DEPRESSION | 1.0 2.0 | Total

-----------+---------------+-------

1.0 | 7 0 | 7

> 100.0% 0.0% | 100.0%

| 100.0% -1.$% |

2.0 | 0 0 | 0

> -1.$% -1.$% | 0.0%

| 0.0% -1.$% |

-----------+---------------+-------

Total | 7 0 | 7

| 100.0% 0.0% |

V8ESCURREN = 1.0

V15EVENT = 2.0

V3SEX = 1.0

V17FHD = 1.0

V13CONNECT = 2.0

V2AGE = 1.0

| V10ACADEMI |

DEPRESSION | 1.0 2.0 | Total

-----------+---------------+-------

1.0 | 12 0 | 12

> 100.0% 0.0% | 63.2%

| 70.6% 0.0% |

2.0 | 5 2 | 7

> 71.4% 28.6% | 36.8%

| 29.4% 100.0% |

-----------+---------------+-------

Total | 17 2 | 19

| 89.5% 10.5% |

V8ESCURREN = 1.0

V15EVENT = 2.0

V3SEX = 1.0

V17FHD = 1.0

V13CONNECT = 2.0

V2AGE = 2.0

| V10ACADEMI |

DEPRESSION | 1.0 2.0 | Total

-----------+---------------+-------

1.0 | 7 3 | 10

> 70.0% 30.0% | 47.6%

| 58.3% 33.3% |

2.0 | 5 6 | 11

> 45.5% 54.5% | 52.4%

| 41.7% 66.7% |

-----------+---------------+-------

Total | 12 9 | 21

| 57.1% 42.9% |

V8ESCURREN = 1.0

V15EVENT = 2.0

V3SEX = 1.0

V17FHD = 2.0

V13CONNECT = 1.0

V2AGE = 1.0

| V10ACADEMI |

DEPRESSION | 1.0 2.0 | Total

-----------+---------------+-------

1.0 | 7 1 | 8

> 87.5% 12.5% | 25.8%

| 31.8% 11.1% |

2.0 | 15 8 | 23

> 65.2% 34.8% | 74.2%

| 68.2% 88.9% |

-----------+---------------+-------

Total | 22 9 | 31

| 71.0% 29.0% |

V8ESCURREN = 1.0

V15EVENT = 2.0

V3SEX = 1.0

V17FHD = 2.0

V13CONNECT = 1.0

V2AGE = 2.0

| V10ACADEMI |

DEPRESSION | 1.0 2.0 | Total

-----------+---------------+-------

1.0 | 13 0 | 13

> 100.0% 0.0% | 33.3%

| 40.6% 0.0% |

2.0 | 19 7 | 26

> 73.1% 26.9% | 66.7%

| 59.4% 100.0% |

-----------+---------------+-------

Total | 32 7 | 39

| 82.1% 17.9% |

V8ESCURREN = 1.0

V15EVENT = 2.0

V3SEX = 1.0

V17FHD = 2.0

V13CONNECT = 2.0

V2AGE = 1.0

| V10ACADEMI |

DEPRESSION | 1.0 2.0 | Total

-----------+---------------+-------

1.0 | 33 1 | 34

> 97.1% 2.9% | 38.6%

| 52.4% 4.0% |

2.0 | 30 24 | 54

> 55.6% 44.4% | 61.4%

| 47.6% 96.0% |

-----------+---------------+-------

Total | 63 25 | 88

| 71.6% 28.4% |

V8ESCURREN = 1.0

V15EVENT = 2.0

V3SEX = 1.0

V17FHD = 2.0

V13CONNECT = 2.0

V2AGE = 2.0

| V10ACADEMI |

DEPRESSION | 1.0 2.0 | Total

-----------+---------------+-------

1.0 | 23 3 | 26

> 88.5% 11.5% | 26.3%

| 34.3% 9.4% |

2.0 | 44 29 | 73

> 60.3% 39.7% | 73.7%

| 65.7% 90.6% |

-----------+---------------+-------

Total | 67 32 | 99

| 67.7% 32.3% |

V8ESCURREN = 1.0

V15EVENT = 2.0

V3SEX = 2.0

V17FHD = 1.0

V13CONNECT = 1.0

V2AGE = 1.0

| V10ACADEMI |

DEPRESSION | 1.0 2.0 | Total

-----------+---------------+-------

1.0 | 5 2 | 7

> 71.4% 28.6% | 87.5%

| 83.3% 100.0% |

2.0 | 1 0 | 1

> 100.0% 0.0% | 12.5%

| 16.7% 0.0% |

-----------+---------------+-------

Total | 6 2 | 8

| 75.0% 25.0% |

V8ESCURREN = 1.0

V15EVENT = 2.0

V3SEX = 2.0

V17FHD = 1.0

V13CONNECT = 1.0

V2AGE = 2.0

| V10ACADEMI |

DEPRESSION | 1.0 2.0 | Total

-----------+---------------+-------

1.0 | 0 0 | 0

> -1.$% -1.$% | 0.0%

| -1.$% 0.0% |

2.0 | 0 1 | 1

> 0.0% 100.0% | 100.0%

| -1.$% 100.0% |

-----------+---------------+-------

Total | 0 1 | 1

| 0.0% 100.0% |

V8ESCURREN = 1.0

V15EVENT = 2.0

V3SEX = 2.0

V17FHD = 1.0

V13CONNECT = 2.0

V2AGE = 1.0

| V10ACADEMI |

DEPRESSION | 1.0 2.0 | Total

-----------+---------------+-------

1.0 | 2 0 | 2

> 100.0% 0.0% | 33.3%

| 50.0% 0.0% |

2.0 | 2 2 | 4

> 50.0% 50.0% | 66.7%

| 50.0% 100.0% |

-----------+---------------+-------

Total | 4 2 | 6

| 66.7% 33.3% |

V8ESCURREN = 1.0

V15EVENT = 2.0

V3SEX = 2.0

V17FHD = 1.0

V13CONNECT = 2.0

V2AGE = 2.0

| V10ACADEMI |

DEPRESSION | 1.0 2.0 | Total

-----------+---------------+-------

1.0 | 2 0 | 2

> 100.0% 0.0% | 22.2%

| 25.0% 0.0% |

2.0 | 6 1 | 7

> 85.7% 14.3% | 77.8%

| 75.0% 100.0% |

-----------+---------------+-------

Total | 8 1 | 9

| 88.9% 11.1% |

V8ESCURREN = 1.0

V15EVENT = 2.0

V3SEX = 2.0

V17FHD = 2.0

V13CONNECT = 1.0

V2AGE = 1.0

| V10ACADEMI |

DEPRESSION | 1.0 2.0 | Total

-----------+---------------+-------

1.0 | 3 0 | 3

> 100.0% 0.0% | 18.8%

| 21.4% 0.0% |

2.0 | 11 2 | 13

> 84.6% 15.4% | 81.3%

| 78.6% 100.0% |

-----------+---------------+-------

Total | 14 2 | 16

| 87.5% 12.5% |

V8ESCURREN = 1.0

V15EVENT = 2.0

V3SEX = 2.0

V17FHD = 2.0

V13CONNECT = 1.0

V2AGE = 2.0

| V10ACADEMI |

DEPRESSION | 1.0 2.0 | Total

-----------+---------------+-------

1.0 | 9 1 | 10

> 90.0% 10.0% | 30.3%

| 33.3% 16.7% |

2.0 | 18 5 | 23

> 78.3% 21.7% | 69.7%

| 66.7% 83.3% |

-----------+---------------+-------

Total | 27 6 | 33

| 81.8% 18.2% |

V8ESCURREN = 1.0

V15EVENT = 2.0

V3SEX = 2.0

V17FHD = 2.0

V13CONNECT = 2.0

V2AGE = 1.0

| V10ACADEMI |

DEPRESSION | 1.0 2.0 | Total

-----------+---------------+-------

1.0 | 12 3 | 15

> 80.0% 20.0% | 26.8%

| 32.4% 15.8% |

2.0 | 25 16 | 41

> 61.0% 39.0% | 73.2%

| 67.6% 84.2% |

-----------+---------------+-------

Total | 37 19 | 56

| 66.1% 33.9% |

V8ESCURREN = 1.0

V15EVENT = 2.0

V3SEX = 2.0

V17FHD = 2.0

V13CONNECT = 2.0

V2AGE = 2.0

| V10ACADEMI |

DEPRESSION | 1.0 2.0 | Total

-----------+---------------+-------

1.0 | 10 2 | 12

> 83.3% 16.7% | 17.4%

| 20.8% 9.5% |

2.0 | 38 19 | 57

> 66.7% 33.3% | 82.6%

| 79.2% 90.5% |

-----------+---------------+-------

Total | 48 21 | 69

| 69.6% 30.4% |

V8ESCURREN = 2.0

V15EVENT = 1.0

V3SEX = 1.0

V17FHD = 1.0

V13CONNECT = 1.0

V2AGE = 1.0

| V10ACADEMI |

DEPRESSION | 1.0 2.0 | Total

-----------+---------------+-------

1.0 | 3 0 | 3

> 100.0% 0.0% | 100.0%

| 100.0% -1.$% |

2.0 | 0 0 | 0

> -1.$% -1.$% | 0.0%

| 0.0% -1.$% |

-----------+---------------+-------

Total | 3 0 | 3

| 100.0% 0.0% |

V8ESCURREN = 2.0

V15EVENT = 1.0

V3SEX = 1.0

V17FHD = 1.0

V13CONNECT = 1.0

V2AGE = 2.0

| V10ACADEMI |

DEPRESSION | 1.0 2.0 | Total

-----------+---------------+-------

1.0 | 0 0 | 0

> -1.$% -1.$% | 0.0%

| 0.0% 0.0% |

2.0 | 4 3 | 7

> 57.1% 42.9% | 100.0%

| 100.0% 100.0% |

-----------+---------------+-------

Total | 4 3 | 7

| 57.1% 42.9% |

V8ESCURREN = 2.0

V15EVENT = 1.0

V3SEX = 1.0

V17FHD = 1.0

V13CONNECT = 2.0

V2AGE = 1.0

| V10ACADEMI |

DEPRESSION | 1.0 2.0 | Total

-----------+---------------+-------

1.0 | 4 0 | 4

> 100.0% 0.0% | 57.1%

| 66.7% 0.0% |

2.0 | 2 1 | 3

> 66.7% 33.3% | 42.9%

| 33.3% 100.0% |

-----------+---------------+-------

Total | 6 1 | 7

| 85.7% 14.3% |

V8ESCURREN = 2.0

V15EVENT = 1.0

V3SEX = 1.0

V17FHD = 1.0

V13CONNECT = 2.0

V2AGE = 2.0

| V10ACADEMI |

DEPRESSION | 1.0 2.0 | Total

-----------+---------------+-------

1.0 | 0 0 | 0

> -1.$% -1.$% | 0.0%

| 0.0% 0.0% |

2.0 | 3 2 | 5

> 60.0% 40.0% | 100.0%

| 100.0% 100.0% |

-----------+---------------+-------

Total | 3 2 | 5

| 60.0% 40.0% |

V8ESCURREN = 2.0

V15EVENT = 1.0

V3SEX = 1.0

V17FHD = 2.0

V13CONNECT = 1.0

V2AGE = 1.0

| V10ACADEMI |

DEPRESSION | 1.0 2.0 | Total

-----------+---------------+-------

1.0 | 5 0 | 5

> 100.0% 0.0% | 71.4%

| 100.0% 0.0% |

2.0 | 0 2 | 2

> 0.0% 100.0% | 28.6%

| 0.0% 100.0% |

-----------+---------------+-------

Total | 5 2 | 7

| 71.4% 28.6% |

V8ESCURREN = 2.0

V15EVENT = 1.0

V3SEX = 1.0

V17FHD = 2.0

V13CONNECT = 1.0

V2AGE = 2.0

| V10ACADEMI |

DEPRESSION | 1.0 2.0 | Total

-----------+---------------+-------

1.0 | 3 0 | 3

> 100.0% 0.0% | 75.0%

| 75.0% -1.$% |

2.0 | 1 0 | 1

> 100.0% 0.0% | 25.0%

| 25.0% -1.$% |

-----------+---------------+-------

Total | 4 0 | 4

| 100.0% 0.0% |

V8ESCURREN = 2.0

V15EVENT = 1.0

V3SEX = 1.0

V17FHD = 2.0

V13CONNECT = 2.0

V2AGE = 1.0

| V10ACADEMI |

DEPRESSION | 1.0 2.0 | Total

-----------+---------------+-------

1.0 | 5 3 | 8

> 62.5% 37.5% | 29.6%

| 22.7% 60.0% |

2.0 | 17 2 | 19

> 89.5% 10.5% | 70.4%

| 77.3% 40.0% |

-----------+---------------+-------

Total | 22 5 | 27

| 81.5% 18.5% |

V8ESCURREN = 2.0

V15EVENT = 1.0

V3SEX = 1.0

V17FHD = 2.0

V13CONNECT = 2.0

V2AGE = 2.0

| V10ACADEMI |

DEPRESSION | 1.0 2.0 | Total

-----------+---------------+-------

1.0 | 6 5 | 11

> 54.5% 45.5% | 45.8%

| 46.2% 45.5% |

2.0 | 7 6 | 13

> 53.8% 46.2% | 54.2%

| 53.8% 54.5% |

-----------+---------------+-------

Total | 13 11 | 24

| 54.2% 45.8% |

V8ESCURREN = 2.0

V15EVENT = 1.0

V3SEX = 2.0

V17FHD = 1.0

V13CONNECT = 1.0

V2AGE = 2.0

| V10ACADEMI |

DEPRESSION | 1.0 2.0 | Total

-----------+---------------+-------

1.0 | 1 0 | 1

> 100.0% 0.0% | 25.0%

| 33.3% 0.0% |

2.0 | 2 1 | 3

> 66.7% 33.3% | 75.0%

| 66.7% 100.0% |

-----------+---------------+-------

Total | 3 1 | 4

| 75.0% 25.0% |

V8ESCURREN = 2.0

V15EVENT = 1.0

V3SEX = 2.0

V17FHD = 1.0

V13CONNECT = 2.0

V2AGE = 1.0

| V10ACADEMI |

DEPRESSION | 1.0 2.0 | Total

-----------+---------------+-------

1.0 | 0 0 | 0

> -1.$% -1.$% | 0.0%

| 0.0% 0.0% |

2.0 | 3 1 | 4

> 75.0% 25.0% | 100.0%

| 100.0% 100.0% |

-----------+---------------+-------

Total | 3 1 | 4

| 75.0% 25.0% |

V8ESCURREN = 2.0

V15EVENT = 1.0

V3SEX = 2.0

V17FHD = 1.0

V13CONNECT = 2.0

V2AGE = 2.0

| V10ACADEMI |

DEPRESSION | 1.0 2.0 | Total

-----------+---------------+-------

1.0 | 1 0 | 1

> 100.0% 0.0% | 14.3%

| 16.7% 0.0% |

2.0 | 5 1 | 6

> 83.3% 16.7% | 85.7%

| 83.3% 100.0% |

-----------+---------------+-------

Total | 6 1 | 7

| 85.7% 14.3% |

V8ESCURREN = 2.0

V15EVENT = 1.0

V3SEX = 2.0

V17FHD = 2.0

V13CONNECT = 1.0

V2AGE = 2.0

| V10ACADEMI |

DEPRESSION | 1.0 2.0 | Total

-----------+---------------+-------

1.0 | 2 0 | 2

> 100.0% 0.0% | 50.0%

| 100.0% 0.0% |

2.0 | 0 2 | 2

> 0.0% 100.0% | 50.0%

| 0.0% 100.0% |

-----------+---------------+-------

Total | 2 2 | 4

| 50.0% 50.0% |

V8ESCURREN = 2.0

V15EVENT = 1.0

V3SEX = 2.0

V17FHD = 2.0

V13CONNECT = 2.0

V2AGE = 1.0

| V10ACADEMI |

DEPRESSION | 1.0 2.0 | Total

-----------+---------------+-------

1.0 | 1 1 | 2

> 50.0% 50.0% | 15.4%

| 14.3% 16.7% |

2.0 | 6 5 | 11

> 54.5% 45.5% | 84.6%

| 85.7% 83.3% |

-----------+---------------+-------

Total | 7 6 | 13

| 53.8% 46.2% |

V8ESCURREN = 2.0

V15EVENT = 1.0

V3SEX = 2.0

V17FHD = 2.0

V13CONNECT = 2.0

V2AGE = 2.0

| V10ACADEMI |

DEPRESSION | 1.0 2.0 | Total

-----------+---------------+-------

1.0 | 1 0 | 1

> 100.0% 0.0% | 4.2%

| 10.0% 0.0% |

2.0 | 9 14 | 23

> 39.1% 60.9% | 95.8%

| 90.0% 100.0% |

-----------+---------------+-------

Total | 10 14 | 24

| 41.7% 58.3% |

V8ESCURREN = 2.0

V15EVENT = 2.0

V3SEX = 1.0

V17FHD = 1.0

V13CONNECT = 1.0

V2AGE = 1.0

| V10ACADEMI |

DEPRESSION | 1.0 2.0 | Total

-----------+---------------+-------

1.0 | 4 0 | 4

> 100.0% 0.0% | 100.0%

| 100.0% -1.$% |

2.0 | 0 0 | 0

> -1.$% -1.$% | 0.0%

| 0.0% -1.$% |

-----------+---------------+-------

Total | 4 0 | 4

| 100.0% 0.0% |

V8ESCURREN = 2.0

V15EVENT = 2.0

V3SEX = 1.0

V17FHD = 1.0

V13CONNECT = 1.0

V2AGE = 2.0

| V10ACADEMI |

DEPRESSION | 1.0 2.0 | Total

-----------+---------------+-------

1.0 | 1 0 | 1

> 100.0% 0.0% | 50.0%

| 50.0% -1.$% |

2.0 | 1 0 | 1

> 100.0% 0.0% | 50.0%

| 50.0% -1.$% |

-----------+---------------+-------

Total | 2 0 | 2

| 100.0% 0.0% |

V8ESCURREN = 2.0

V15EVENT = 2.0

V3SEX = 1.0

V17FHD = 1.0

V13CONNECT = 2.0

V2AGE = 1.0

| V10ACADEMI |

DEPRESSION | 1.0 2.0 | Total

-----------+---------------+-------

1.0 | 0 0 | 0

> -1.$% -1.$% | 0.0%

| 0.0% 0.0% |

2.0 | 1 1 | 2

> 50.0% 50.0% | 100.0%

| 100.0% 100.0% |

-----------+---------------+-------

Total | 1 1 | 2

| 50.0% 50.0% |

V8ESCURREN = 2.0

V15EVENT = 2.0

V3SEX = 1.0

V17FHD = 1.0

V13CONNECT = 2.0

V2AGE = 2.0

| V10ACADEMI |

DEPRESSION | 1.0 2.0 | Total

-----------+---------------+-------

1.0 | 1 0 | 1

> 100.0% 0.0% | 10.0%

| 50.0% 0.0% |

2.0 | 1 8 | 9

> 11.1% 88.9% | 90.0%

| 50.0% 100.0% |

-----------+---------------+-------

Total | 2 8 | 10

| 20.0% 80.0% |

V8ESCURREN = 2.0

V15EVENT = 2.0

V3SEX = 1.0

V17FHD = 2.0

V13CONNECT = 1.0

V2AGE = 1.0

| V10ACADEMI |

DEPRESSION | 1.0 2.0 | Total

-----------+---------------+-------

1.0 | 1 0 | 1

> 100.0% 0.0% | 14.3%

| 20.0% 0.0% |

2.0 | 4 2 | 6

> 66.7% 33.3% | 85.7%

| 80.0% 100.0% |

-----------+---------------+-------

Total | 5 2 | 7

| 71.4% 28.6% |

V8ESCURREN = 2.0

V15EVENT = 2.0

V3SEX = 1.0

V17FHD = 2.0

V13CONNECT = 1.0

V2AGE = 2.0

| V10ACADEMI |

DEPRESSION | 1.0 2.0 | Total

-----------+---------------+-------

1.0 | 2 1 | 3

> 66.7% 33.3% | 33.3%

| 33.3% 33.3% |

2.0 | 4 2 | 6

> 66.7% 33.3% | 66.7%

| 66.7% 66.7% |

-----------+---------------+-------

Total | 6 3 | 9

| 66.7% 33.3% |

V8ESCURREN = 2.0

V15EVENT = 2.0

V3SEX = 1.0

V17FHD = 2.0

V13CONNECT = 2.0

V2AGE = 1.0

| V10ACADEMI |

DEPRESSION | 1.0 2.0 | Total

-----------+---------------+-------

1.0 | 7 0 | 7

> 100.0% 0.0% | 15.6%

| 28.0% 0.0% |

2.0 | 18 20 | 38

> 47.4% 52.6% | 84.4%

| 72.0% 100.0% |

-----------+---------------+-------

Total | 25 20 | 45

| 55.6% 44.4% |

V8ESCURREN = 2.0

V15EVENT = 2.0

V3SEX = 1.0

V17FHD = 2.0

V13CONNECT = 2.0

V2AGE = 2.0

| V10ACADEMI |

DEPRESSION | 1.0 2.0 | Total

-----------+---------------+-------

1.0 | 3 3 | 6

> 50.0% 50.0% | 12.5%

| 14.3% 11.1% |

2.0 | 18 24 | 42

> 42.9% 57.1% | 87.5%

| 85.7% 88.9% |

-----------+---------------+-------

Total | 21 27 | 48

| 43.8% 56.3% |

V8ESCURREN = 2.0

V15EVENT = 2.0

V3SEX = 2.0

V17FHD = 1.0

V13CONNECT = 2.0

V2AGE = 1.0

| V10ACADEMI |

DEPRESSION | 1.0 2.0 | Total

-----------+---------------+-------

1.0 | 0 0 | 0

> -1.$% -1.$% | 0.0%

| -1.$% 0.0% |

2.0 | 0 2 | 2

> 0.0% 100.0% | 100.0%

| -1.$% 100.0% |

-----------+---------------+-------

Total | 0 2 | 2

| 0.0% 100.0% |

V8ESCURREN = 2.0

V15EVENT = 2.0

V3SEX = 2.0

V17FHD = 1.0

V13CONNECT = 2.0

V2AGE = 2.0

| V10ACADEMI |

DEPRESSION | 1.0 2.0 | Total

-----------+---------------+-------

1.0 | 0 0 | 0

> -1.$% -1.$% | 0.0%

| -1.$% 0.0% |

2.0 | 0 1 | 1

> 0.0% 100.0% | 100.0%

| -1.$% 100.0% |

-----------+---------------+-------

Total | 0 1 | 1

| 0.0% 100.0% |

V8ESCURREN = 2.0

V15EVENT = 2.0

V3SEX = 2.0

V17FHD = 2.0

V13CONNECT = 1.0

V2AGE = 1.0

| V10ACADEMI |

DEPRESSION | 1.0 2.0 | Total

-----------+---------------+-------

1.0 | 0 0 | 0

> -1.$% -1.$% | 0.0%

| 0.0% 0.0% |

2.0 | 1 1 | 2

> 50.0% 50.0% | 100.0%

| 100.0% 100.0% |

-----------+---------------+-------

Total | 1 1 | 2

| 50.0% 50.0% |

V8ESCURREN = 2.0

V15EVENT = 2.0

V3SEX = 2.0

V17FHD = 2.0

V13CONNECT = 1.0

V2AGE = 2.0

| V10ACADEMI |

DEPRESSION | 1.0 2.0 | Total

-----------+---------------+-------

1.0 | 0 0 | 0

> -1.$% -1.$% | 0.0%

| 0.0% 0.0% |

2.0 | 7 2 | 9

> 77.8% 22.2% | 100.0%

| 100.0% 100.0% |

-----------+---------------+-------

Total | 7 2 | 9

| 77.8% 22.2% |

V8ESCURREN = 2.0

V15EVENT = 2.0

V3SEX = 2.0

V17FHD = 2.0

V13CONNECT = 2.0

V2AGE = 1.0

| V10ACADEMI |

DEPRESSION | 1.0 2.0 | Total

-----------+---------------+-------

1.0 | 2 0 | 2

> 100.0% 0.0% | 4.8%

| 11.8% 0.0% |

2.0 | 15 25 | 40

> 37.5% 62.5% | 95.2%

| 88.2% 100.0% |

-----------+---------------+-------

Total | 17 25 | 42

| 40.5% 59.5% |

V8ESCURREN = 2.0

V15EVENT = 2.0

V3SEX = 2.0

V17FHD = 2.0

V13CONNECT = 2.0

V2AGE = 2.0

| V10ACADEMI |

DEPRESSION | 1.0 2.0 | Total

-----------+---------------+-------

1.0 | 0 1 | 1

> 0.0% 100.0% | 2.1%

| 0.0% 3.6% |

2.0 | 19 27 | 46

> 41.3% 58.7% | 97.9%

| 100.0% 96.4% |

-----------+---------------+-------

Total | 19 28 | 47

| 40.4% 59.6% |

** Beginning Stratified Analysis **

Summary Odds Ratio, N = 1213

+-------------+--------+------+-------------+----------+-------------+----------+-----------+

| DEPRESSION | crude | wt | 95% CI | MH Summ | test for | p-value | nz margin |

| | OR | OR | wt OR | Chi Sq | interaction | | tables |

+-------------+--------+------+-------------+----------+-------------+----------+-----------+

| V10ACADEMI | 3.87 | 2.97 | 2.16 - 4.08 | 44.88 | 34.0927 | 0.9962 | 44/60 |

+-------------+--------+------+-------------+----------+-------------+----------+-----------+

| V8ESCURREN | 2.75 | 2.18 | 1.57 - 3.02 | 22.09 | 16.2960 | 0.9999 | 43/60 |

+-------------+--------+------+-------------+----------+-------------+----------+-----------+

| V15EVENT | 2.51 | 1.99 | 1.54 - 2.57 | 27.39 | 41.6164 | 0.9661 | 40/61 |

+-------------+--------+------+-------------+----------+-------------+----------+-----------+

| V3SEX | 2.05 | 1.95 | 1.47 - 2.59 | 21.54 | 23.8035 | 0.9999 | 40/61 |

+-------------+--------+------+-------------+----------+-------------+----------+-----------+

| V17FHD | 1.97 | 1.85 | 1.35 - 2.54 | 14.60 | 15.1201 | 0.9999 | 44/61 |

+-------------+--------+------+-------------+----------+-------------+----------+-----------+

| V13CONNECT | 2.28 | 1.75 | 1.33 - 2.30 | 16.05 | 30.5828 | 0.9996 | 40/62 |

+-------------+--------+------+-------------+----------+-------------+----------+-----------+

| V2AGE | 1.30 | 1.36 | 1.05 - 1.77 | 5.36 | 30.6512 | 0.9992 | 41/60 |

+-------------+--------+------+-------------+----------+-------------+----------+-----------+
